# Early endostatin treatment inhibits metastatic seeding of murine colorectal cancer cells in the liver and their adhesion to endothelial cells

**DOI:** 10.1038/sj.bjc.6602385

**Published:** 2005-02-08

**Authors:** E A te Velde, A Reijerkerk, D Brandsma, J M Vogten, Y Wu, O Kranenburg, E E Voest, M Gebbink, I H M Borel Rinkes

**Affiliations:** 1 Department of Surgery, University Medical Center Utrecht, Utrecht, The Netherlands; 2 Department of Medical Oncology, University Medical Center Utrecht, Utrecht, The Netherlands; 3 Department of Neurology, University Medical Center Utrecht, Utrecht, The Netherlands; 4 Department of Hematology, University Medical Center Utrecht, Utrecht, The Netherlands

**Keywords:** endostatin, adhesion, colorectal metastases, *in vivo* microscopy, multiphoton laser scanning microscopy

## Abstract

Endostatin, a carboxy-terminal fragment of collagen XVIII, potently inhibits angiogenesis and tumour growth, presumably through induction of apoptosis in endothelial cells and/or inhibition of their migration. Here we have tested how the timing of recombinant human endostatin (rh-E) administration affects its antitumour activity in a liver metastasis model of mouse C26 colorectal carcinoma cells. The effects of rh-E treatment on hepatic tumour load and on early tumour cell seeding were evaluated. Recombinant human endostatin was most effective in reducing intrahepatic tumour growth when administered prior to tumour cell inoculation. Analysis of early tumour cell seeding by using [^125^I]iododeoxyuridine-labelled C26 cells or by *in vivo* microscopy showed that rh-E reduced tumour cell seeding in the liver sinusoids. Recombinant human endostatin did not inhibit tumour growth when administered later than 4 days after tumour injection. Pretreatment of human umbilical vein endothelial cells with rh-E *in vitro* reduced C26 tumour cell adhesion under flow conditions two-fold as assessed by video microscopy and multiphoton laser scanning microscopy. Our results show that rh-E, in addition to antiangiogenic effects, reduces tumour cell adhesion in the liver sinusoids during the very early phases of metastasis formation. These data point towards a previously unknown mode of action of endostatin, that is, its ability to interfere with tumour cell seeding. Such insights may be helpful in the design of trials to improve (surgical) treatment of colorectal carcinoma and liver metastases.

Antiangiogenic drugs are directed against components of the developing vasculature. Based on the working mechanism of these drugs, that is, preventing outgrowth of microscopic tumour deposits, little or no antitumour effects might be anticipated in gross or bulky (metastatic) disease. In the case of established (unresectable) tumours, treatment with antiangiogenic agents will at best lead to disease stabilisation, rather than to complete tumour remission. Furthermore, discontinuation of therapy will allow the tumour or its metastases to resume their outgrowth (Boehm *et al*, 1997). Taken together, initiation of the treatment already during the initial phase of tumour growth can be essential in optimising antiangiogenic therapy.

Endostatin, a naturally occurring fragment of collagen XVIII, is one of the most effective inhibitors of angiogenesis and dramatically reduced tumour growth in several mouse models with no serious side effects observed (Boehm *et al*, 1997; O'Reilly *et al*, 1997; Kisker *et al*, 2001). However, endostatin was ineffective in other studies, and these data have prompted the discussion about the efficacy of endostatin (Marshall, 2002). In a previous report by our group, endostatin treatment resulted in significant antitumour effects in a model of murine colorectal liver metastases (te Velde *et al*, 2002). These effects, as measured 12 days following tumour cell injection by histology, were achieved by administration of endostatin from day 0 until day 12. Others found that the efficacy of endostatin improved when administered before tumour cell injection, as measured by tumour load 21 days following tumour cell injection (Solaun *et al*, 2002). In a pilot study, we have observed that endostatin efficacy depended on the timing of its administration. We were not able to show regression of already established colorectal liver metastases when endostatin treatment was initiated 7 days after tumour cell injection.

To optimise the timing of endostatin administration, it is imperative that its mechanism of action is resolved. So far, endostatin is known to have antiangiogenic properties, that is, its antitumour effects depend on inhibition of developing tumour vasculature. This action has predominantly been ascribed to inhibition of apoptosis and migration of endothelial cells (Yamaguchi *et al*, 1999; Sasaki *et al*, 2002). Many studies point towards an effect of endostatin on the adhesion of endothelial cells to other endothelial cells (Dixelius *et al*, 2002): these inhibitory effects may be mediated by direct blocking of specific integrins (Rehn *et al*, 2001; Furumatsu *et al*, 2002), by disassembly of focal adhesions and actin stress fibres (Wickstrom *et al*, 2001) and/or by antagonising the Wnt pathway (Hanai *et al*, 2002). Based on these studies, we hypothesised that (antiadhesive) antitumour properties of endostatin could play a role even shortly after tumour cell inoculation, before new vessel formation is needed for tumour outgrowth. Very recently, others showed that endostatin affects melanoma cells while metastasising to the liver in an experimental setting (Mendoza *et al*, 2004).

In this study, we have investigated the influence of endostatin on the early spatiotemporal fate of murine colon carcinoma cells metastasising to the liver. We show that administration of recombinant human endostatin (rh-E) prior to tumour cell injection resulted in reduced tumour cell seeding in the liver and in reduced intrahepatic tumour growth. Furthermore, rh-E induced a two-fold decrease of tumour cell adhesion to endothelial cells under flow conditions *in vitro*.

## MATERIALS AND METHODS

### Endostatin

Recombinant human endostatin was produced in *Pichia pastoris* (Courtesy of Entremed, Rockville, USA). In mice, 500 *μ*g was administered by daily subcutaneous (s.c.) injection of 100 *μ*l. Control animals received the equal amount of solvent citrate buffer (17 mM citric acid, 59 mM NaCl, 66 mM Na_2_PO_4_, pH 6.2). For the *in vitro* experiments, three equivalent dosages of endostatin and its control were used, that is, 400, 200 and 100 *μ*g ml^−1^. Only freshly thawed endostatin was used in the experiments presented.

### Tumour cell culture

The murine colon carcinoma cell line C26 was routinely cultured in Dulbecco's modified Eagle's medium (DMEM) supplemented with 10% heat-inactivated fetal calf serum (FCS), 100 U ml^−1^ penicillin and 100 *μ*g ml^−1^ streptomycin in a 10% CO_2_ environment. Cell viability was determined by Trypan blue staining. Confluent cultures were harvested by brief trypsinisation (0.05 trypsin in 0.02% EDTA), and resuspended in phosphate-buffered saline (PBS) to a final concentration of 10^6^ × 1.0 cells ml^−1^ (unless stated otherwise).

### Endothelial cell culture

Human vascular endothelial cells (HUVECs) were isolated from human umbilical veins. Cells were cultured in endothelial basal medium (EBM) containing EGM-2 medium (Clonetics, Bio Whitaker, San Diego, CA, USA) supplemented with 5% FBS, gentamicin, amphotericin B, hydrocortisone, ascorbic acid and the following growth factors: VEGF, bFGF, hEGF and IGF-1. Cells of passage two were seeded on fibronectin-coated glass coverslips and grown until confluency.

### *In vivo* liver metastases model

Balb/C male mice (aged 10 weeks) purchased from Harlan (Leicestershire, Great Britain) were housed under standard conditions and allowed food and water *ad libitum*. Colorectal liver metastases were induced in all mice as follows (te Velde *et al*, 2002). Mice were anaesthetised intraperitoneally with fentanyl citrate/fluanisone (0.3 mg per mouse; Janssen-Cilag, Brussels, Belgium) and midazolamchloride (12.5 mg per mouse; Roche, Brussels, Belgium). Through a left lateral flank incision, C26 colorectal carcinoma cells were injected into the spleen parenchyma. In case mice were allowed to survive 24 h, the spleen was removed after 10 min to avoid intrasplenic tumour growth. All experiments were performed in accordance with the guidelines of the Animal Welfare Committee of the UMC Utrecht, The Netherlands, as well as with those of the UKCCCR guidelines on the treatment of animals, and all animals received humane care.

### Histological analysis

To determine the effects on tumour load of different schemes of endostatin administration, mice (*n*=24) were randomly assigned to the following experimental groups: endostatin treatment starting 2 h before tumour cell injection and 8 h, 4 days and 7 days after tumour cell injection. The treatment was discontinued on days 4 and 12. Lower concentrations of tumour cells were used for treatment groups until day 19 (10^5^ × 1.0 ml^−1^). For histological analysis of tumour burden, the mice were killed on days 7, 12 or 19 (*n*=3 per group). The livers were harvested, formaldehyde fixed and embedded in paraffin. Intrahepatic tumour load was scored as the hepatic replacement area (HRA), the percentage of hepatic tissue having been taken up by metastatic tumour cells. This was assessed on nonsequential haematoxylin- and eosin-stained sections by semiautomated stereology (Leica-Q-Prodit system, Leica Microsystems, Rijswijk, The Netherlands) using a four points grid overlaid on 100 fields per slide at a magnification of × 40.

### Intrahepatic detection of radiolabelled tumour cells

To determine the spatiotemporal fate of tumour cells metastasising to the liver, we used radiolabelled tumour cells. To prepare labelled cells for intrasplenic injection, C26 tumour cells were cocultured with 1 *μ*Ci ml^−1^ [^125^I]iododeoxyuridine (Amersham Biosciences, Weert, The Netherlands) in DMEM containing 5% FCS for 72 h at 37°C in 10% CO_2_ environment. The cells were washed in PBS, detached with trypsin-EDTA, washed with serum-containing media and twice with PBS and resuspended to a final concentration of 10^6^ × 5.3 cells ml^−1^ in PBS.

Intrasplenic injection of [^125^I]iododeoxyuridine-labelled tumour cells was followed by hepatectomy at *t*=15 min and 1 h (*n*=5 per group per time point per experiment). The resected livers were rinsed in 70% ethanol. Radioisotope levels were measured by a gamma counter and expressed as percentage of the input dose (Cpma). The input dose was determined by measuring two 100-*μ*l aliquots of PBS containing [^125^I]iododeoxyuridine-labelled tumour cells in parallel with the liver samples (100%).

### *In vivo* microscopy

To examine the fate of tumour cells in the liver after intrasplenic injection by *in vivo* microscopy, C26 cells were labelled with a fluorescent probe, carboxyfluorescein succinyl ester (CFSE; Molecular Probes, Leiden, The Netherlands). Tumour cells were incubated for 15 min at 37°C with 20 *μ*l of 4% CFSE in PBS containing 0.1% bovine serum albumin (BSA). Cells were centrifuged, resuspended in DMEM and incubated for 30 min at 37°C. Cells were then centrifuged and resuspended for intrasplenic injection. A fraction of tumour cells was cultured in parallel to confirm fluorescence in 99% of the cells.

At 15 min and 1 h after intrasplenic injection of C26 tumour cells, intravital fluorescence microscopy was performed using a Nikon TE-300 inverted microscope (Uvikon, The Netherlands) equipped with a filter set for fluorescein (excitation 450–490 nm, emission >515 nm). Images were registered with a charge coupled device camera (Exwave HAD, Sony, The Netherlands) and recorded with an s-VHS VCR (Panasonic). Using a high magnification × 40 lens, 10–15 randomly selected fields were chosen in each animal (*n*=20 mice); images were recorded for 1 min and were analysed off-line. At indicated time points, the extent of metastasis was measured as number of tumour cells per high-power field (hpf).

### Tumour cell migration assay

The C26 tumour cells were grown to 75–90% confluency. After washing, the cells were trypsinised, pelleted and resuspended in DMEM/0.1% BSA. The cells were labelled *in situ* with 10 *μ*M Calcein-AM in DMEM for 15 min at 37°C and subsequently added to 8 *μ*m FALCON® HTS FluoroBlok™ Cell Culture Insert (Becton, Dickinson and Company, Franklin Lakes, NJ, USA), at a density of 100 000 cells per insert. We used DMEM supplemented with 10% FCS as a chemoattractant in the lower wells, while DMEM/0.1% BSA was added to the control wells. Different concentrations of endostatin (100 and 400 *μ*g ml^−1^) or equal concentrations of control buffer were added. The inserts were incubated for 15 min, 1 h, 4 h and 24 h at 37°C. The number of migrated cells was measured using a PE Biosystems CytoFluor 4000 plate reader at a gain setting of 54.

### Tumour cell death assay

Tumour cells were exposed to different concentrations of endostatin (400, 200 and 100 *μ*g ml^−1^) in culture medium for 24 h. The total pool of adherent and detached cells was obtained by collecting the detached cells. The remaining adherent cells were trypsinised and added to the detached cells in the medium. One half of the cells were stained with 0.02% Trypan blue and the percentage of dead (Trypan blue-positive) cells was assessed using a Bûrker glass counter chamber. Duplicate samples were analysed. In addition, the other half of the cells was fixed in the culture medium using 3.7% formaldehyde and analysed.

### Tumour cell adhesion assay under flow conditions

The effects of endostatin on the *in vitro* adhesion of C26 tumour cells to stimulated HUVECs were assessed under flow conditions. The HUVECs were stimulated with TNF-*α* (PeproTech, London, England) at a concentration of 10 ng ml^−1^ for 3 h before tumour cell perfusion. At 2 h before perfusion, the stimulated HUVECs were incubated with endostatin at a concentration of 400 *μ*g ml^−1^, an equal concentration of BSA or citrate buffer only.

First, the adhesion of C26 colon carcinoma cells (10^6^ × 4.0 cells ml^−1^, 1% FCS, 25 mM Hepes buffer) to endostatin (*n*=7) *vs* citrate buffer (*n*=8)-treated TNF-*α*-stimulated HUVECs was investigated using a modified form of a transparent perfusion chamber (Brandsma *et al*, 2002). Data from two independent experiments were pooled. The microchamber has a slit height of 0.2 mm and a width of 2 mm and contains a plug on which a coverslip (18 mm × 18 mm) with confluent HUVECs was mounted. C26 colon carcinoma cells were aspirated from a reservoir through the perfusion chamber with a Harvard syringe pump (Harvard Apparatus, South Natic, MA, USA). In this way, the flow rate through the chamber could be controlled precisely. The wall shear stress (*t*) was 50 mPa. During the perfusion, the flow chamber was mounted on a microscope stage (DM RXE, Leica, Wetzlar, Germany), equipped with a B/W CCD video camera (Sanyo, Osaka, Japan) connected to a VHS video recorder. Perfusion experiments were recorded real time on a videotape. Video images were analysed off-line for the number of adherent C26 colon carcinoma cells with a Quantimet 570C image analysis system (Leica Cambridge, Cambridge, UK). The number of surface-adherent C26 cells per mm^2^ was measured after 5 min of perfusion at a minimum of 25 fields (total surface ⩾1.0 mm^2^) using custom-made software developed in Optimas 6.1 (Media Cybernetics Systems, Silver Spring, MD, USA).

Second, adhesion of C26 colon carcinoma cells to HUVECs was determined in whole blood (Sixma *et al*, 1998). The HUVECs were seeded on glass coverslips of size 60 × 24 mm and grown to confluency. They were perfused in a small parallel-plate perfusion chamber with a slit height of 0.1 mm and a slit width of 2 mm corresponding with flow rates of 15 *μ*l min^−1^ (shear rate 10 s). Fresh blood from healthy donors was anticoagulated with 1/10 volume of 150 U ml^−1^ Orgaran (a low-molecular-weight heparinoid (LMWH); Organon, Oss, The Netherlands). Blood was prewarmed at 37°C for 10 min and was then drawn through the perfusion chamber by a Harvard infusion pump (pump 22, model 2400-004; Natick, MA, USA). Tumour cells (10^6^ × 6.0 ml^−1^) were incubated with 200 *μ*g ml^−1^ dihydroethidium (3,8-diamino-5,6-dihydro-5-ethyl-6-phenylphenanthridine 2,7-diamino-10-ethyl-9-phenyl-9,10-dihydrophenanthridine hydroethidine). Aliquots of 15 *μ*l tumour cells in 150 *μ*l blood were used per perfusion. Real-time images were recorded using a VCR system and were analysed off-line and the number of adhered tumour cells per mm^2^ confluent HUVEC layer was calculated. After perfusion, full blood perfused coverslips were fixed in a mixture of 2% paraformaldehyde and 0.2% glutaraldehyde in 0.1 mol l^−1^ phosphate buffer (pH 7.4). Staining of cytoskeletal actin was performed by Phalloidin-TRITC. Hoechst 33342 was used for blue nuclear staining, and anti-fibrinogen was stained green (primary antibody rabbit anti-human anti-fibrinogen). The coverslips were mounted by fluorescence medium, FluorSave™ (Calbiochem 345789, La Jolla, CA, USA). The stained coverslips were analysed using multiphoton laser scanning microscopy.

### Statistical analysis

For comparison of the endostatin treatment and the controls, the independent sample *t*-test was used. Data were given as mean±s.e.m. and were considered significant when *P*<0.05.

## RESULTS

### Histological analysis of liver metastases

**Figure 1:**
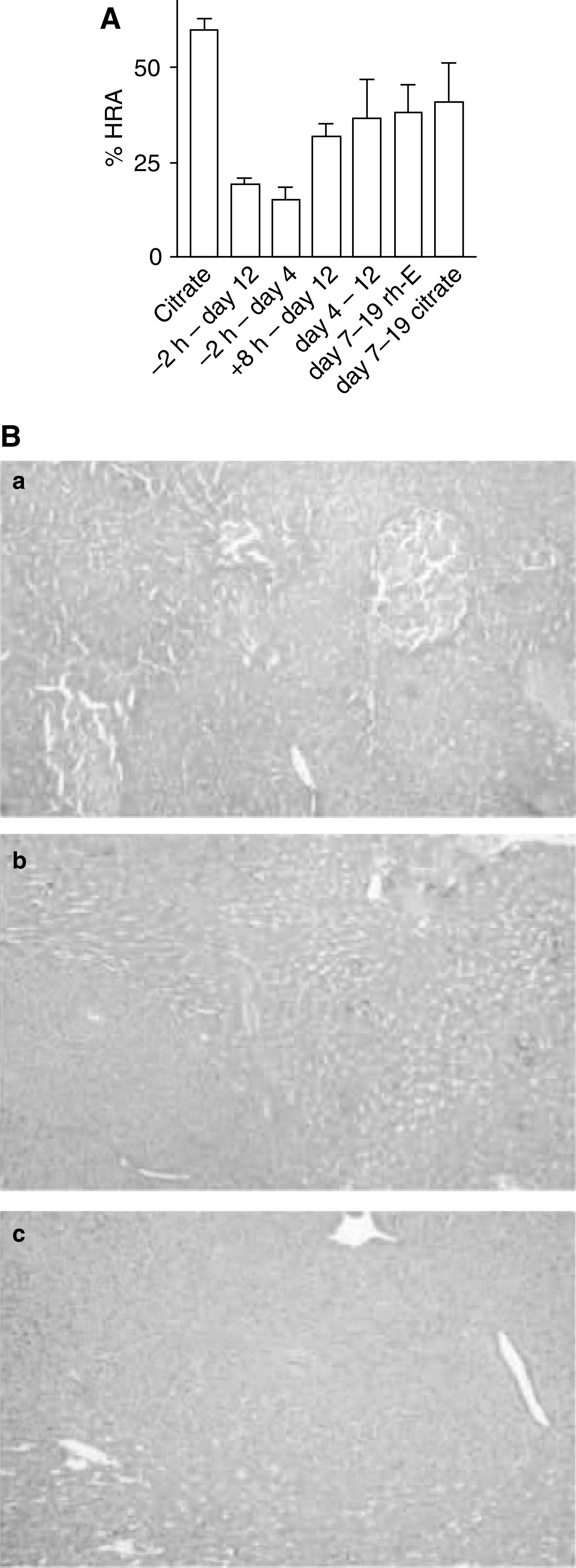
(**A**) Endostatin (rh-E) therapy is most efficacious when started prior to tumour cell seeding. All mice were inoculated with tumour cells on *t*_0_. Recombinant human endostatin or control citrate buffer was given in different time schemes as indicated and the intrahepatic tumour load (HRA) for all different treatment groups was assessed on day 12. Data are plotted as means±s.e.m. (**B**) Reduced therapeutic efficacy of rh-E treatment on established tumours. (a) Haematoxylin- and eosin-stained sections from a liver 7 days after intrasplenic injection of tumour cells just prior to the start of treatment, showing three small intrahepatic tumour lesions (dark nodules). (b) Tumour-bearing liver 19 days after tumour cell injection treated with citrate buffer. (c) Tumour-bearing liver 19 days after tumour cell injection treated with rh-E. The tumours continued to grow under rh-E treatment. For quantification, see (**A**).

Intrahepatic arrest and outgrowth of circulating tumour cells is a multistep process. Mice were injected with C26 tumour cells in the spleen to induce liver metastases and treated with endostatin at different time points. After 12 days, livers were removed and analysed for the extent of replacement of liver tissue by tumour cells (HRA). On day 12, the HRA of the controls (buffer treated) was 60±2.9 (Figure 1A). The HRA in mice that were treated with endostatin from 2 h prior to tumour cell injection until killing was the lowest: 19±2.1% (*P*<0.001). When treatment was started 2 h before tumour cell injection and replaced by citrate buffer from day 4 until day 12, the HRA was still significantly decreased (15±3.5%, *P*=0.001). When treatment was started 8 h after tumour cell injection and continued until day 12, there was a significant antitumour effect as well (HRA 32±3.4%, *P*=0.002). However, initiation of the treatment on day 4 or later after tumour cell injection did not result in antitumour efficacy as reflected by HRA (36.7±10.1%, *P*=0.139). Treatment from day 7 until day 19 did not reduce tumour load and resulted in an HRA of 38.3±7.3 *vs* 41±10% in the citrate buffer-treated controls (*P*=0.8) (Figure 1A and B).

### Intrahepatic detection of radiolabelled tumour cells

**Figure 2:**
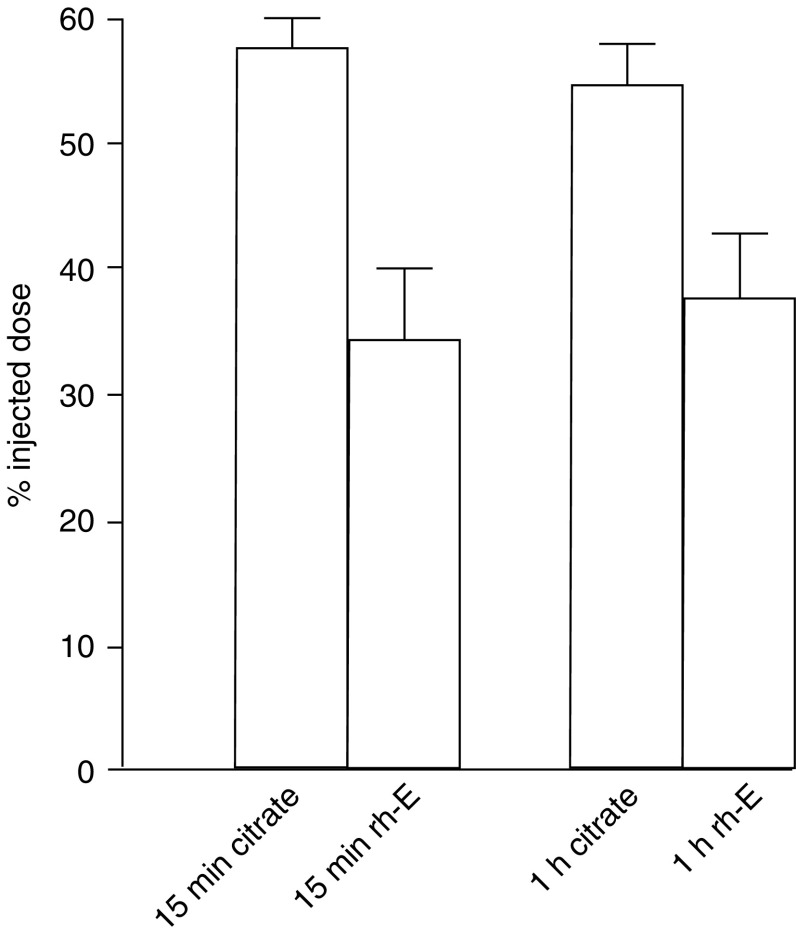
Radioactive labelled tumour cells in the liver, 15 min and 1 h after intrasplenic injection, as measured by gamma counter and represented as percentage of injected [^125^I]iododeoxyuridine-labelled tumour cells. At 15 min after tumour cell injection in the spleen, the percentage of injected dose in the liver was 34.4±5.6% in the mice that were treated with rh-E 2 h prior to tumour cell injection, *vs* 57.4±2.5% in the controls (*P*=0.001). After 1 h, these percentages had not significantly changed.

The above results suggest that endostatin acts at a very early stage of metastasis formation. To investigate the initial arrest of circulating C26 tumour cells in the liver of endostatin-pretreated mice, we measured radiolabelled tumour cells after injection into the spleen. Livers were collected at 15 and 60 min after tumour cell injection and the radioactivity was subsequently determined. Data from two independent experiments were pooled. In all, 50% of the intrasplenic injected tumour cells were arrested in the liver during the first 15 min, as measured by radioactivity. Endostatin pretreatment reduced the percentage of injected dose in the liver to 34.4±5.6% at 15 min after tumour cell injection *vs* 57.4±2.5% in the controls (*P*=0.001; Figure 2). When radiolabelled cells in the liver were measured after 1 h, these percentages had not significantly changed (37.6±5.2 *vs* 54.8±3.2%).

### *In vivo* microscopy

**Figure 3:**
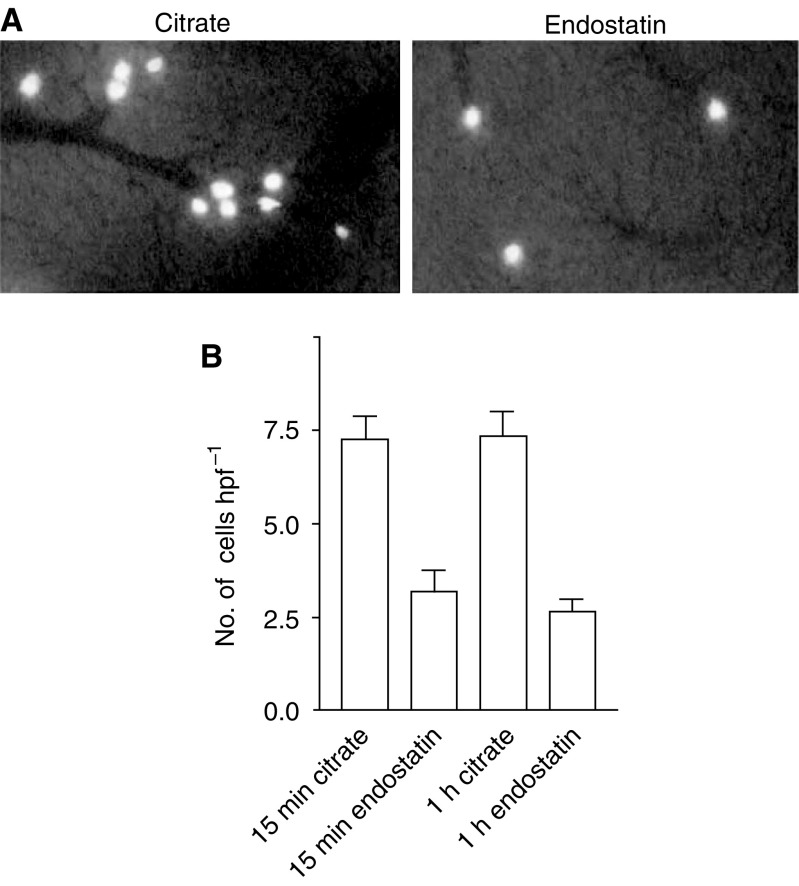
(**A**) Intravital microscopy images recorded 15 min after injection of fluorescent tumour cells into the spleen of a control liver and a liver after 2 h rh-E pretreatment. (**B**) Number of fluorescent tumour cells that are present in the liver per hpf as measured by intravital microscopy 15 min and 1 h after intrasplenic injection. Recombinant human endostatin reduced the number of arrested tumour cells in the liver by 56% (*P*<0.001). No differences were observed within either treatment group between the two time points (controls 15 min *vs* 1 h *P*=0.93; endostatin 15 min *vs* 1 h *P*=0.39).

Initial tumour cell arrest in the liver was studied in more detail by *in vivo* microscopy. C26 tumour cells were fluorescently labelled with calcein and injected into the spleens of recipient mice. Tumour cells reaching the liver were easily discernable from the hepatic tissue (Figure 2). Two independent experiments were pooled (*n*=20). At 15 min after injection of tumour cells into the spleen, the control mice showed a mean of 7.3±0.6 fluorescent tumour cells per hpf (Figure 3A), whereas in the mice that were treated with endostatin 2 h prior to injection, less arrested intrahepatic cells (3.2±0.5 cells hpf^−1^) were counted (*P*<0.001; Figure 3B). When images were made 1 h after tumour cell injection, this reduction was unchanged (endostatin-treated livers 2.7±0.3 arrested cells per hpf *vs* controls 7.4±0.7 cells per hpf, *P*<0.001). No differences were observed within either treatment group between the two time points 15 min and 1 h after tumour cell injection (controls 15 min *vs* 1 h *P*=0.93; endostatin 15 min *vs* 1 h *P*=0.39).

### Tumour cell assay

Significant Calcein-AM labelled C26 chemotaxis was not detected until 2 h of incubation. The migration of endostatin-treated as compared to the control tumour cells was similar at all time points measured. At the time of maximal migration – at 4 h – the lowest concentration of endostatin resulted in a fluorescent signal of the migrated cells of 6165±926 *vs* 5620±765 in the controls (*P*=0.6). The highest concentration of endostatin did not influence the migration of tumour cells either (5562±1018 *vs* 5268±623 in the controls, *P*=0.8). There was no direct effect of endostatin on cell death of C26 murine colon carcinoma cells (data not shown).

### Tumour cell adhesion assay under flow conditions

**Figure 4:**
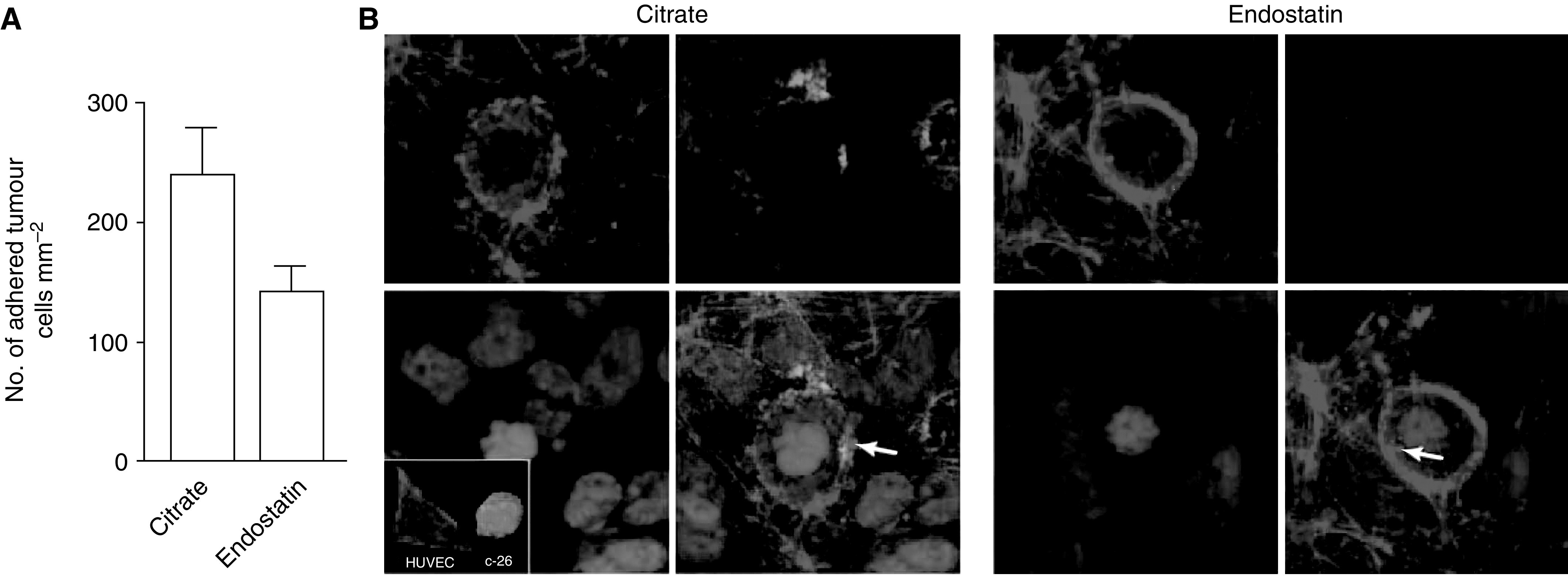
Recombinant human endostatin reduces tumour cell adhesion under flow conditions. (**A**) C26 cells were allowed to adhere to a confluent layer of TNF-*α*-stimulated HUVECs under flow conditions for 5 min and this was recorded on a videotape. The number of adherent tumour cells per mm^2^ was determined by off-line analysis of the video images. Recombinant human endostatin reduced the number of adhered tumour cells to stimulated HUVECs when compared to citrate buffer control by 41% (*P*=0.04). (**B**) Immunofluorescence and multiphoton laser scanning microscopy visualised C26 tumour cell adhesion to HUVECs. After perfusion with tumour cells in full blood, the coverslips were fixed and stained with Hoechst (blue) to stain nuclei, Phalloidin-TRITC (red) to stain filamentous actin and anti-fibrinogen (green) to visualise the extracellular matrix. Platelets are indicated by arrows.

Since endostatin treatment decreased early intrahepatic tumour cell arrest and did not directly influence tumour cell death or migration, we investigated tumour cell–endothelial cell interactions. Fluorescent-labelled tumour cells were perfused over a confluent layer of endothelial cells. Pretreatment (2 h) with endostatin of stimulated HUVECs led to a more than two-fold decrease of tumour cell adhesion under flow conditions (103±18 adhered cells mm^−2^) as compared to pretreatment with control buffer (180±15 adhered cells mm^−2^, *P*=0.007). Shear forces did not induce rolling of the C26 cells, but rather a direct tethering, followed by firm tumour cell adhesion under both control and endostatin conditions. Bovine serum albumin did not inhibit adhesion of tumour cells to HUVECs (data not shown). In concordance, after perfusion with full blood, the adhesion of C26 cells to HUVECs was inhibited with endostatin pretreatment (143±20 adhered cells mm^−2^) *vs* controls (239±41 adhered cells mm^−2^) (*P*=0.0231). Multiphoton laser scanning microscopy visualised the adhesion of tumour cells, surrounded by platelets, to endothelial cells (Figure 4). Reduced tumour cell adhesion by endostatin pretreatment was not associated with detectable changes in tumour or endothelial cell morphology or viability, nor with reduced platelet adhesion.

## DISCUSSION

Several mechanisms have been proposed that might explain the antiangiogenic activity of endostatin (Yamaguchi *et al*, 1999; Rehn *et al*, 2001; Wickstrom *et al*, 2001; Dixelius *et al*, 2002; Furumatsu *et al*, 2002; Hanai *et al*, 2002; Sasaki *et al*, 2002). However, it is unclear whether and to what extent these activities contribute to early antiangiogenic and antimetastatic effects. Undisturbed early tumour cell metastasis is a multistep process that requires intravascular arrest of tumour cells, as well as their adhesion to endothelial cells, followed by transendothelial migration. All of these aforementioned steps – in theory – can be potential targets by which early metastasis formation is inhibited.

In this study, we show that early treatment with endostatin led to inhibition of the number of tumour cells arrested in the liver within 15 min after intrasplenic tumour cell injection, which in turn resulted in enhanced antitumour efficacy. *In vitro*, endostatin neither affected the migration nor caused cell death of the tumour cells. There was, however, a strong inhibitory effect of endostatin pretreatment on the adhesion of tumour cells to endothelial cells under flow conditions. These data cannot be explained by classical ‘antiangiogenesis’ activity. Therefore, our studies provide evidence for a novel mechanism of endostatin action, in addition to its direct effects on angiogenesis and endothelial cells.

*In vitro* experiments mainly focused on endostatin and endothelial cell–cell adhesion revealed an integrin-dependent interaction (Rehn *et al*, 2001; Furumatsu *et al*, 2002). Possibly our findings demonstrating that endostatin inhibits adhesion of tumour cells to endothelial cells and diminishes intrahepatic tumour cell arrest are integrin mediated. This explanation is supported by the findings of Rehn *et al*, who demonstrated that endostatin interacts with *α*_5_*β*_1_, *α*_v_*β*_3_ and *α*_v_*β*_5_ integrins on the surface of HUVECs. Others found that endostatin activity is mediated by an integrin-dependent inhibition of adhesion of endothelial cells to collagen I (Furumatsu *et al*, 2002). Indeed, the recent study on the effects of endostatin on experimental melanoma cells showed that VCAM-1 plays an important role in adhesion of these cells (Mendoza *et al*, 2004). Interestingly, in a study on the mapping of endostatin binding sites in intact human tissues, endostatin was predominantly found to bind to blood vessels (Chang *et al*, 1999). Inhibitory effects of tumour cell adhesion to HUVEC by endostatin are not likely to be explained by a direct effect of endostatin on TNF-*α* upregulation, since this pathway was observed not to be influenced by endostatin (Yin *et al*, 2002).

Furthermore, the ability of endostatin to bind to the proteoglycan heparin (O'Reilly *et al*, 1997) points to a possible involvement of heparin sulphate proteoglycans (HSPGs) in endostatin activity. These are expressed on the cell surface or present in the extracellular matrix (ECM) and mediate the adhesion of cells to other cells or ECM (Ma and Geng, 2000; Engbring *et al*, 2002). Indeed, heparin and heparan sulphate can inhibit experimental tumour metastasis (reviewed in Engelberg, 1999).

On the other hand, one cannot rule out that alternative steps to adhesion could be of importance in early metastasis. Intravascular tumour cell arrest, through direct interactions of tumour cells with components of the blood, can mediate dissemination. Tumour cell arrest can be affected by fibrin deposition (Palumbo and Degen, 2001), as well as platelets–tumour cell interaction (Honn *et al*, 1992). We could speculate that the binding of platelets to the tumour cells is inhibited, possibly mediated by integrins as well. The relative contribution of fibrinogen-, platelet- and endothelial cell-mediated interactions with tumour cells will have to be elucidated in *in vivo* experiments using mice that lack components of the fibrinolytic system.

As in a previous study, we found that endostatin is effective against colorectal liver metastases (te Velde *et al*, 2002). Solaun *et al* showed that the antiangiogenic mechanism (i.e. endothelial cell apoptosis) was selective for sinusoidal-type metastases, in which the neovasculature originating from sinusoidal endothelium cells was targeted by endostatin. Differences in the tumour's microenvironment are known to determine antitumour efficacy of the treatment (Fidler, 1978, 1999) The precursor of endostatin, the long form of collagen XVIII, is almost exclusively found in the liver (Saarela *et al*, 1998) and mainly expressed by hepatocytes (Schuppan *et al*, 1998; Lietard *et al*, 2000). Continuous capillaries contain both XV and XVIII collagen, but fenestrated capillaries (e.g. liver sinusoids, glomeruli, lung alveoli and splenic sinusoids) express only type XVIII (Tomono *et al*, 2002). In our hands, endostatin had no effect on subcutaneous tumours, possibly because the outgrowth of these tumours does not depend on cell seeding (data not shown; see also Schmitz *et al*, 2004). We could speculate that endostatin action in the liver is mediated by dominant-negative competition with its precursor. Such insights into organ specificity might be useful in the design of future clinical trials. Furthermore, since we found optimal antitumour effects even as early as within 15 min after intrasplenic tumour cell injection, endostatin prophylaxis might prove very promising to augment surgical treatment of colorectal carcinoma and liver metastases. Even in no-touch surgery, preventing metastatic seeding and combined with mesenterical vessel ligation, 12.5% of patients have detectable tumour spill in the circulation (Sales *et al*, 1999). Overall, as many as 75% of colorectal cancer patients develop liver metastases (Pickren *et al*, 1982). In this respect, it is worth mentioning that clinical trials in patients with established metastatic disease have shown that endostatin, although well tolerated, generated marginal tumour responses (Eder *et al*, 2002; Herbst *et al*, 2002; Thomas *et al*, 2003). Others also found that the efficacy of endostatin improved when administered before tumour cell injection, as measured by tumour load 21 days following tumour cell injection (Solaun *et al*, 2002). Bergers *et al* (1999) correspondingly showed that endostatin, among others, was capable of treating early stages of cancer. Finally, *in vivo* endostatin gene transfection with a cationic lipid inhibited the outgrowth of fibrosarcoma cells as pulmonary metastases and this was most effective when treatment was started prior to tumour cell inoculation (Yano *et al*, 2004). Taken together, it seems likely that endostatin efficacy will be optimal when administered during the early stages of the disease. Therefore, the antitumour activity of endostatin should be further tested in patients with minimal or microscopic metastatic disease. *In vitro* studies provide evidence to support an early effect of endostatin: disassembly of focal adhesions and actin stress fibres in endothelial cells *in vitro* was described to be already visible 1 h after endostatin administration (Wickstrom *et al*, 2001). In addition, early-response genes responsible for cell–matrix interactions were downregulated rapidly after endostatin treatment (Shichiri and Hirata, 2001).

In conclusion, 2-h pretreatment *in vivo* with endostatin inhibits intrahepatic tumour growth, mainly due to early (<15 min) inhibition of tumour cell seeding in the liver. It also induces a two-fold decrease of tumour cell adhesion to endothelial cells under flow conditions. This appears to be a novel, nonangiogenesis-related working mechanism of endostatin, possibly mediated by integrin-dependent adhesion of tumour cells to endothelial cells. These data can be helpful in the design of future clinical trials to augment surgical treatment of colorectal carcinoma and liver metastases.
